# Decomposition of sediment-oil-agglomerates in a Gulf of Mexico sandy beach

**DOI:** 10.1038/s41598-019-46301-w

**Published:** 2019-07-11

**Authors:** Ioana Bociu, Boryoung Shin, Wm. Brian Wells, Joel E. Kostka, Konstantinos T. Konstantinidis, Markus Huettel

**Affiliations:** 1 Department of Earth, Ocean and Atmospheric Science, Florida State University, 117N Woodward Ave, Tallahassee, FL 32306 USA; 2 School of Earth & Atmospheric Sciences, Georgia Institute of Technology, 310 Ferst Drive, Atlanta, GA 30332-0230 USA; 3 School of Biology, Georgia Institute of Technology, 310 Ferst Drive, Atlanta, GA 30332-0230 USA; 4 School of Civil and Environmental Engineering, Georgia Institute of Technology, 790 Atlantic Drive, Atlanta, GA 30332-0355 USA

**Keywords:** Pollution remediation, Marine chemistry, Marine chemistry

## Abstract

Sediment-oil-agglomerates (SOA) are one of the most common forms of contamination impacting shores after a major oil spill; and following the Deepwater Horizon (DWH) accident, large numbers of SOAs were buried in the sandy beaches of the northeastern Gulf of Mexico. SOAs provide a source of toxic oil compounds, and although SOAs can persist for many years, their long-term fate was unknown. Here we report the results of a 3-year *in-situ* experiment that quantified the degradation of standardized SOAs buried in the upper 50 cm of a North Florida sandy beach. Time series of hydrocarbon mass, carbon content, n-alkanes, PAHs, and fluorescence indicate that the decomposition of golf-ball-size DWH-SOAs embedded in beach sand takes at least 32 years, while SOA degradation without sediment contact would require more than 100 years. SOA alkane and PAH decay rates within the sediment were similar to those at the beach surface. The porous structure of the SOAs kept their cores oxygen-replete. The results reveal that SOAs buried deep in beach sands can be decomposed through relatively rapid aerobic microbial oil degradation in the tidally ventilated permeable beach sand, emphasizing the role of the sandy beach as an aerobic biocatalytical reactor at the land-ocean interface.

## Introduction

After the 2010 Deepwater Horizon explosion, currents and waves moved approximately 22,000 tons of spilled MC252-oil^1^ to the northeastern Gulf of Mexico shoreline^2,3^, where this oil polluted an estimated 965 km of sandy beaches^4–6^. Oil deposited on beaches bound to sand, forming sediment-oil-agglomerates (SOA). At the beach surface, photooxidation and mechanical stress contributed to the degradation of exposed SOAs^7–9^. Lateral sand movement buried SOAs as deep as 70 cm in the beach^10,11^, and the degradation of this buried oil was dominated by microbial decomposition^12–14^. Many sedimentary microorganisms are capable to degrade petroleum hydrocarbons^15–17^. Biodegradation typically begins with the decomposition of the lighter saturated and aromatic hydrocarbons, and results in an increase in polar hydrocarbon compounds generated through the oxidation process^18,19^. Alkanes (Alk) comprise the largest portion of crude oil by mass^20^, while the polar fractions and polycyclic aromatic hydrocarbons (PAH), present at lower concentrations, are critical due to their potential toxicity^21,22^.

Embedded petroleum hydrocarbons can persist in beach sediments for years to decades^23,24^, posing a potential hazard to the environment and humans. To prevent adverse impacts, buried SOAs^25^ were mechanically extracted from Florida sandy beaches after the Deepwater Horizon accident^11^, but despite extensive cleanup efforts, all oil could not be removed^5,6,26^. SOAs persisted in the sand, with <10 cm diameter being the most common size^27^.

Quantitative information on the degradation rates of SOAs embedded in Gulf of Mexico beach sands was not available, partly because the continuous washing up of SOAs onto the beach (e.g. as released from natural and anthropogenic sources and submerged oil mats^5,28,29^) makes the determination of the exact time point when SOAs became buried in the beach difficult. This raised the question how long this beach oil contamination from the DWH accident would persist.

To address this question, a three-year *in-situ* experiment was initiated that quantified the degradation of standardized MC525-SOAs (sSOAs) buried in a Pensacola sandy beach. The use of sSOAs allowed following the degradation of a known source material that was buried at a known time point, and direct comparison of sSOA composition at the times of burial and after excavation. sSOA decomposition rates were quantified through mass and carbon losses, changes in saturated and aromatic hydrocarbon concentrations, as well as changes in sSOA fluorescence. The resulting decay rates were used to estimate how long the oil would persist in the beach. A companion study by Shin *et al*. (submitted to Scientific Reports) elucidates the dynamics of the sedimentary microbial communities mediating sSOA degradation over time.

## Methods

### Study site

The study took place at the municipal beach at Pensacola Beach/Florida (30°20′55.8″N, 87°02′52.2″W, Fig. S1) from October 2010 to December 2013. This beach was oil contaminated after the DWH accident but the experiment took place in a section of the beach that was not reached by the oil. The uncontaminated beach sediment is composed of well-sorted quartz sands (grain size median 449 μm, first quartile 363 μm, and third quartile 550 μm, porosity 49%) with very low organic (0.065% ± 1.251%, determined by Loss On Ignition (LOI) at 550 °C) and inorganic carbon contents (0.011% ± 0.009%, LOI at 950 °C). Annual average air temperature was 19.9 °C (Fig. S2a). In the upper 50 cm of the beach, temperatures remained above 25 °C from May to September and did not drop below 10 °C during the rest of the year^10^. The moisture content of the beach sand above the spring tide high water line ranged from 0.2 to 6.0% (Fig. S2b). Oxygen concentrations in the sand decreased with sediment depth but remained above 50% oxygen air saturation within the upper 50 cm of the beach (Fig. S2c).

### Standardized sediment-oil-agglomerate (sSOA) arrays and surface residual balls (sSRB)

**Figure 1:**
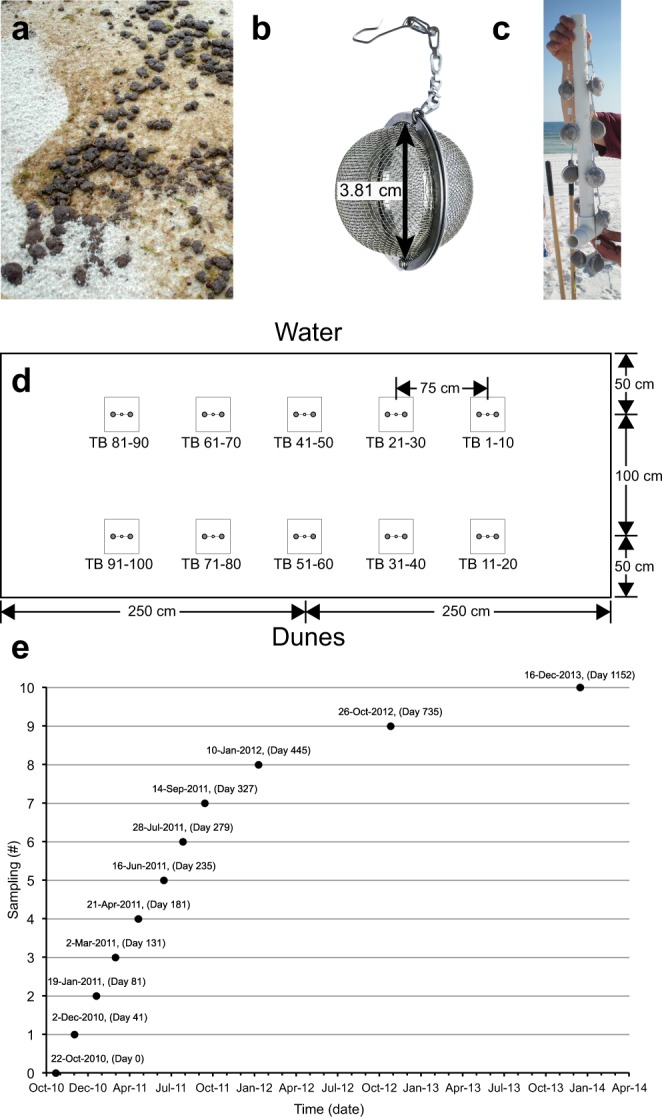
Preparation, burial and retrieval of the experimental sSOAs. (**a**) SOAs at Pensacola Beach that were collected to produce the homogenized oil-sand material that was filled into stainless steel tea balls like the one shown in (**b**) The tea ball could be opened for filling, and a chain attached to its solid metal flanges permitted attaching the filled tea ball to the PVC pipe. (**c**) Array of 10 sSOA-filled mesh balls attached to the PVC pipe prior to burial. (**d**) Dimensions of the burial plot and locations of the sites, where the individual arrays were buried. (**e**) Time line of the experiment: Initial installation of sSOAs and sSRBs was in October 2010. sSOA arrays were retrieved from December 2010 to December 2013, (2 Dec10, 19 Jan 11, 2 Mar 11, 21 Apr 11, 16 Jun 11, 28 Jul 11, 14 Sep 11, 10 Jan 12, 26 Oct 12, 16 Dec 13), sSRBs in April 2011 and July 2011. Days elapsed are shown in parentheses as “Day #”.

Approximately 5 kg of freshly formed MC252-SOAs (1–5 cm diameter, Fig. 1a) were collected at the study site on June 30^th^ 2010, one week after the oil reached the Florida coast. The SOAs were pooled and homogenized. One hundred stainless steel mesh-balls (tea-balls, 3.81 cm inner diameter, Fig. 1b) were filled with 33.7 g (SD = 3.2) of the compacted homogenized material, producing standardized SOAs, termed “sSOA”. These sSOAs contained 4.8 g (14.3% by weight) petroleum hydrocarbons, 0.9 g (2.6%) water, and 28.0 g (83.1%) sand. The mesh size (1 mm × 1 mm openings) of the mesh-balls allowed quasi-unrestricted exchange of gases and solutes considering the average pore diameter (~0.04 mm diam.) of the sand. Five pairs of sSOAs were attached to a 50 cm long vertical PVC pipe (1.3 cm diameter), such that pairs were positioned a 10, 20, 30, 40 and 50 cm sediment depth (Fig. 1c). Ten of such sSOA arrays were buried in the supralittoral dry beach, approximately 1 m above the spring-tide high water line and about 1.5 m above the average beach groundwater level (assessed by digging down to the groundwater table), out of the reach of seawater and groundwater (Fig. 1d). In the center of the area, 10 standardized surface residual balls (sSRBs) were affixed to the sediment surface to allow comparison of petroleum hydrocarbon degradation of surface and buried sediment-oil-agglomerates (sSRB: 34 g sSOA material wrapped in transparent nylon gauze (1 mm mesh) producing cylindrical agglomerates (2 cm diameter, 5 cm length)). The 10 sSRBs were attached with nylon fishing line to two PVC anchors such that the sSRBs would rest horizontally on the sediment surface.

The sSOA arrays were deployed on October 22^nd^, 2010, and retrieved at seven monthly to bimonthly intervals for approximately one year and thereafter at three larger intervals until the final sampling on 16 December 2013 (Fig. 1e). At each time point, one array with 10 sSOAs was retrieved. sSRBs could only be recovered in April 2011 and July 2011 because all other sSRBs disappeared (most likely removed by curious vertebrates). Retrieved sSOAs and sSRBs were stored in a −20 °C freezer. Subsamples of the sSOAs were sent to Georgia Institute of Technology for microbial community analysis. Because only three data points are available for the sSRB time series, reported sSRB decay rate constants are best estimates produced by forcing a first order exponential decay function through the data points.

### Determination of sSOA mass and fluorescence loss

Adhering sand grains were dusted off the retrieved sSOAs prior to weighing, and photographing. The fluorescence of the sSOA surface was measured with a Waltz™ fluorometer at an excitation wave length of 650 nm and collecting emission wavelengths at 710 nm^30^ (more details of the method in Supplemental Materials).

### Carbon analysis

2.0 mg aliquots from each sSOA were placed in tin cups and analyzed for total carbon content in a Carlo Erba Elemental Analyzer (EA).

### Petroleum hydrocarbon analysis

A ~200 mg aliquot of each sSOA was weighed and extracted in an accelerated pressurized solvent extractor (Buchi SpeedExtractor E-914) according to the US EPA Method 3545A modified for in-cell extract clean-up (more details in Supplemental Materials). Extraction recovery averaged 102% (1 SD = 31.7). The resulting 40 mL DCM-solution was concentrated in a Buchi Syncore Analyst and transferred to n-hexane producing a 1 mL hydrocarbon extract. The extracts were analyzed on an Agilent 7890A gas chromatograph J&W (Agilent Select PAH GC Column (30 m × 0.25 mm × 0.15 µm)) equipped with an Agilent 7000 Triple Quadrupole mass spectrometer. Alkanes from 10 to 40 carbons in length (Table S1) and 36 aromatic hydrocarbons (Table S2) were evaluated. The PAHs included the 16 US EPA priority compounds and 11 additional PAHs listed in the International Agency for Research on Cancer (IARC). First order kinetics was applied for calculating decay rates. The Limit of Detection (LOD) ranged from 3 to 300 ng cm^−3^ for *n*-alkanes and 0.2 to 14 ng cm^−3^ for PAHs. Tables S1 and S2 list the MRM method parameters after optimization with the Agilent method optimization software.

### Oxygen concentration in sSOAs

Oxygen time series were used to determine whether the core of sSOAs would become oxygen depleted over time. A calibrated oxygen fiber optode (Pyroscience™, 430 µm Fiber diameter) was mounted either through a narrow hole (sSOAs 1 and 2) or a cut (SOA 3) such that the sensor tip was located in the center of the sSOA. Oxygen concentrations then were measured using a Pyroscience™ FireStingO2 oxygen meter over a period of 3 months. During this period, the temperature was constant (22 °C) and the moisture ranged from 60 to 80%.

### Reference rSOAs

To assess the contribution of the beach environment (e.g. temperature, moisture, nutrients, contact to sand with bacteria) to the sSOA and sSRB degradation, their decomposition was compared to that of sSOA material incubated in the laboratory at constant temperature (22 °C) in the dark. Ten aliquots (1.4 ± 0.3 g) of the sSOA material, termed rSOA, were placed onto polystyrene dishes, weighed and then stored in a closed cabinet. During the incubation, the relative humidity in the lab varied between 60 and 70%. The ten samples were weighted on March 12, 2011, August 27, 2011, February 5, 2017 and August 18, 2018.

### Statistical analyses

T statistics were applied for testing the significance of slopes and the two tailed student t-test with a 95% confidence interval was used to compare data after assessment of normality with the Kolmogorov-Smirnov test.

More details regarding the methods are provided in the Supplemental Materials. The data are available through the GoMRI GRIIDC data server at https://data.gulfresearchinitiative.org/.

## Results and Discussion

**Figure 2:**
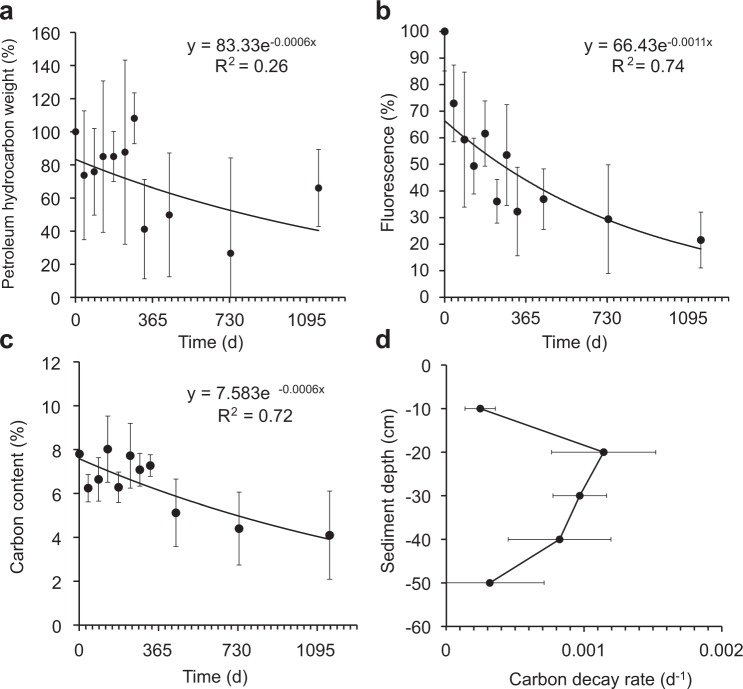
Decreases in sSOA mass, fluorescence and carbon during the *in-situ* experiment and carbon decay rates (**a**) Decrease of the mass of the hydrocarbons in the sSOAs (starting weight = 100%). The relatively large variability was caused by the sand grains adhering to the sSOAs. (**b**) Decrease of average sSOA fluorescence. (**c**) sSOA carbon loss over time. (**d**) sSOA carbon content decay at different sediment depths. Error bars depict one standard deviation.

Losses of mass, carbon, fluorescence, and changes in petroleum hydrocarbons documented the decomposition of the oil compounds in the sand-oil-agglomerates over the three-year duration of the experiment (Fig. 2). Their consistency changed from soft and cohesive to hard and brittle, with a soil-like appearance at the end. sSOA coloration turned from brown to black (Fig. S3a–c), and fluorescence, typically associated with aromatic hydrocarbons^29,30^, decreased (Fig. 2b, Fluorescence_(t)_ (%) = 66.43e^−0.0011d^, R² = 0.75, p = 0.0037). Petroleum hydrocarbon mass in the sSOAs decreased over 3 years from 4.82 g (1 SD = 0.43 g) to 1.95 g (1 SD = 1.70 g) and at a similar rate as the total carbon content of the sSOAs (Fig. 2a,c, Petroleum hydrocarbon mass_(t)_ (%) = 83.40e^−0.00063d^, R^2^ = 0.26, Carbon content_(t)_ (%) = 7.583e^−0.00057d^, R^2^ = 0.72, p < 0.0009). Since the organic and inorganic carbon content of the uncontaminated beach sand that makes up more than 80% of the SOA material is small (<0.1% of sediment weight), the observed carbon decrease in the sSOAs can be attributed to the loss of crude oil compounds. After 1 year, the sSOAs had lost 19% of their carbon, after 2 years 34%, and 48% by the end of the experiment (1152 days).

**Figure 3:**
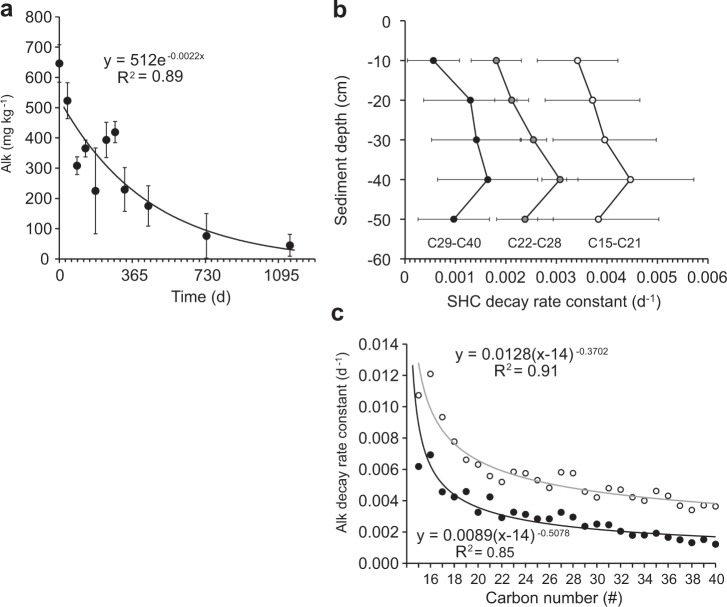
Alkane degradation in the sSOAs during the experiment. (**a**) Degradation of the total *n*-alkanes. (**b**) Decay rate constants for *n*-alkanes at the different sediment depths. Rate constants are averaged according to carbon numbers 15–21, 22–28 and 29–40. (**c**) Decay rate constants for C_15_-C_40_ *n*-alkanes determined for the initial half-year (open symbols, grey line) and the 3-year time period of the experiment (solid symbols, black line).

Decay rate constants of sSOA carbon increased from the sediment surface to 20 cm depth and declined deeper in the sediment column (Fig. 2d). The relatively high content of low molecular weight compounds in the light sweet MC252 Louisiana crude enhances its degradability^31,32^, and although the oil reaching the shoreline was already moderately weathered^33^, C_18_-C_26_ hydrocarbons comprised the majority of *n*-alkanes in the source material for the sSOAs and sSRBs collected at Pensacola Beach (Figs S4a and S5). Alkanes in this molecular weight range exhibited rapid biodegradation in previous studies^34^, and largely due to their depletion, the cumulative concentration of all measured *n*-alkanes (C_15_–C_40_) decreased from 646 mg kg^−1^ (1 SD = 62 mg kg^−1^) in October 2010 to 45 mg kg^−1^ (1 SD = 36 mg kg^−1^) in December 2013, corresponding to a loss of 93% (Fig. 3a, *n*-alkane concentration_(t)_ (mg kg^−1^) = 512e^−0.0022d^, R² = 0.90, p < 0.0001). Independent of carbon number, *n*-alkane decay rates increased with sediment depth, reaching maximum values at 40 cm, and diminishing deeper in the sediment column (Fig. 3b).

Alkanes contributed 98% of the GC/MS amenable petroleum hydrocarbons in the sSOAs. Across the spectrum of *n*-alkanes measured (C_15_-C_40_) at each time point, concentrations in general increased from C_15_ to C_22_ and then decreased toward C_40_ (Figs S4a and S5, Table S3), and initially (Oct 2010) ranged from 1 mg kg^−1^ (C_15_) to 513 mg kg^−1^ (C_22_). Alkane decay rate constants decreased exponentially with carbon number from 0.0062d^−1^ for C_15_ to 0.0012d^−1^ for C_40_ (Fig. 3c, Decay rate constant *k* (d^−1^) = 0.0089*(carbon number-14)^−0.5078^, R² = 0.91). During the first half-year of the experiment, rate constants were on average 2-fold higher than those calculated for the overall 3-year time course (Fig. 3c, Table S4, 0.5 years: 0.0056d^−1^ (1 SD = 0.0022d^−1^), 3 years: 0.0030d^−1^ (1 SD = 0.0014d^−1^)). This short-term to long-term ratio increased linearly from 1.7 for C_15_ to 3.0 for C_40_ (Fig. S4b) suggesting a priming effect^35,36^ of the lighter hydrocarbons in the degradation process of the heavier compounds in the first half year of the experiment.

**Figure 4:**
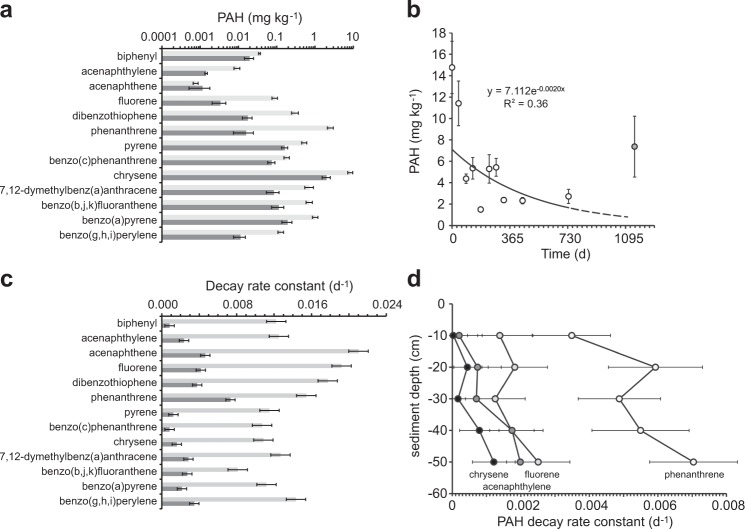
PAH degradation in the sSOAs during the experiment. (**a**) PAH concentrations in the sSOAs at the start of the experiment (light gray) and after 2 years (dark gray). (**b**) Degradation of the total PAHs during the experiment. The PAH concentration of day 1152 (grey fill) was excluded from the regression due to analytical issues. Error bars depict one standard deviation. The dashed line extrapolates the trend based on the 2-year data set. (**c**) PAH decay rate constants averaged over all sediment depths for the initial half-year (light grey bars) and the 2-year period (dark grey bars). Error bars depict standard error. (**d**) Decay rate constants of selected PAHs calculated for the different sediment depths. Error bars depict one standard deviation.

Out of the 27 PAHs evaluated, 13 occurred above the detection limits (biphenyl, acenaphthylene, acenaphthene, fluorene, dibenzothiophene, phenanthrene, pyrene, benzo(c)phenanthrene, chrysene, 7,12-dimethylbenz(a)anthracene, benzo(b,j,k)fluoranthene, benzo(a)pyrene, and benzo(g,h,i)perylene), and contributed about 2.2% of total GC/MS amenable petroleum hydrocarbons. The initial PAH concentrations (1 to 8459 µg kg^−1^, Oct 2010, Fig. 4a, Table S5) were approximately 2 orders of magnitude lower than those of the *n*-alkanes. Chrysene concentrations exceeded those of the other detectable PAHs by one to two orders of magnitude. The average total PAH concentration decreased from 14.8 mg kg^−1^ (1 SD = 2.4 mg kg^−1^) in October 2010 to 2.7 mg kg^−1^ (1 SD = 0.7 mg kg^−1^) in October 2012 (a 82% loss, PAH concentration_(t)_ (mg kg^−1^) = 7.112e^−0.0020d^, R² = 0.36), and thus at a similar rate as the *n*-alkanes. The PAH measurements of the 3-year time point were compromised by analytical issues, but extrapolation of the 2-year trend implies that 90% of the PAHs were decomposed after 3 years (Fig. 4b).

Phenanthrene had the highest 3-year decay rate constant (0.0073d^−1^) and benzo(c)phenanthrene the lowest (0.0008d^−1^), reflecting a decrease of the decay rate with increasing number of benzene rings (Figs 4c and S6). The average 0.5-year PAH rate constant (0.0137d^−1^, 1 SD = 0.0037) exceeded that of the 2-year time course (0.0029d^−1^ (1 SD = 0.0018), Fig. 4c, Table S6). This supports the hypothesis of a “priming effect” during the first half year, when relatively high PAH concentrations and presence of highly degradable oil compounds enhanced overall PAH decomposition^37^. In general, PAH decay increased with increasing sediment depth (Fig. 4d), but in contrast to the *n*-alkanes, the PAH decay rate profiles showed a peak at 20 cm sediment depth (except for biphenyl and acenaphthylene).

**Figure 5:**
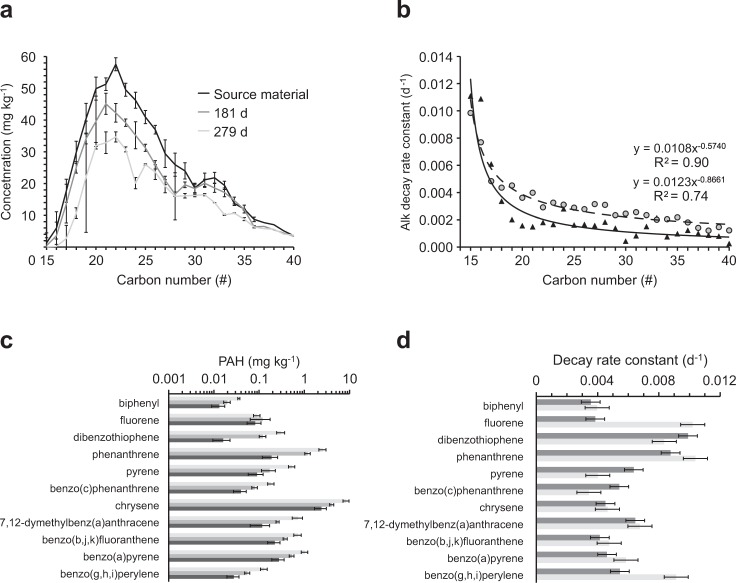
Alkane and PAH degradation in the sSRBs during the experiment. (**a**) Alkane concentrations in the sSRBs at the beginning of the experiment and after 0.5 and 0.75 years. Error bars depict standard deviation. (**b**) Comparison of *n*-alkane decay rate constants in sSRBs (0.75-year decay rates, black triangles, solid line) and sSOAs (0.75-year decay rates, grey circles, dotted line). (**c**) PAH concentrations in the sSRBs at the start of the experiment (light gray) and after 0.5 (medium gray) and 0.75 years (dark gray). Error bars depict one standard deviation. Note logarithmic scaling of the X-axis. (**d**) Comparison of 0.75-year sSRB (dark grey bars) and sSOA (light grey bars) PAH decay rate constants. Error bars depict standard error.

The sSRBs at the beach surface were exposed to different and more variable environmental conditions compared to the buried sSOAs, which influenced alkane and PAH degradation in these agglomerates in different ways (Fig. 5a,c). The decomposition of *n*-alkanes in the sSRBs was slower (Fig. 5a,b, sSRB-Alk: 0.75-year rate: 0.0018d^−1^) than in the sSOAs (sSOA-Alk: 0.0022d^−1^), which may have been caused by the higher average moisture content within the beach. At the beach surface, photolysis can enhance PAH decomposition^9,38^ and accelerate their decay through attack of tertiary carbon atoms that can block biodegradation^39,40^. This process may explain the ~16% larger decay rate constants of the GC/MS-amenable PAHs in the exposed sSRBs (0.0051d^−1^) compared to the respective rates in the buried sSOAs (0.75-year rate: 0.0043d^−1^) during the initial 0.75-year period when sSRBs could be sampled (Fig. 5d). However, due to variability, the differences between sSRBs and sSOAs in the decay rates of the individual GC/MS amenable *n*-alkanes and PAHs were statistically not significant (t-test: p = 0.449). At the calculated rates, the sSRBs would lose approximately 85% of their *n*-alkanes and 99% of their PAHs within 3 years. These decay rates are similar to those reported by Aeppli *et al*.^18^ who found 79% (1 SD = 2%) depletion of the saturated and aromatic fractions in MC252 SRBs collected between 2011 and 2017 along the Gulf of Mexico, suggesting that the decomposition of the agglomerates progressed at similar pace at the different Gulf beaches.

The relatively high rates of petroleum hydrocarbon degradation we observed were supported by the aerobic environment within the sediment-oil-agglomerates. Because the buried sSOAs were protected from photodegradation and mechanical stress, their degradation can be attributed to microbial decomposition as reflected by oxygen consumption within the sSOAs and within oil layers buried in the beach^10,11^. The oxygen measurements in the center of sSOAs recorded an initial decrease from ~100% oxygen air saturation (sSOA1: 99.73% (1 SD = 0.21%), sSOA2: 99.68% (1 SD = 0.17%), sSOA3: 99.59% (1 SD = 0.14%)) to ~98% air saturation within 7 days (sSOA1: 98.54% (1 SD = 0.16%), sSOA2: 98.28% (1 SD = 0.32%), sSOA3: 98.34% (1 SD = 0.13%)). Afterward, concentrations remained relatively constant (sSOA1: 98.50% (1 SD = 0.02%), sSOA2: 98.27% (1 SD = 0.05%), sSOA3: 98.18% (1 SD = 0.47)). The initial insertion of the oxygen sensor allowed oxygen in the center of the sSOAs to equilibrate with air resulting in oxygen air saturation in the pore space at the beginning of the measurements. Oxygen concentration then dropped to a level governed by the balance of microbial oxygen consumption and oxygen supply, and thereafter remained relatively steady. These steady concentrations in the core of the sSOAs, despite ongoing aerobic degradation processes, imply a continuous supply of oxygen. Owing to their relatively high sand content (Deepwater Horizon SOAs: 84–88%^26^, sSOAs: 86%), the sediment-oil-agglomerates have a porous structure (Fig. S3e,f) that allows gas penetration, as confirmed by permeability measurements conducted on SOAs and heavily oiled sand layers at Pensacola Beach^10^. Even after burial of oil, the Pensacola beach sediment remained permeable to gases, which allowed air exchange in the sand through tidal pumping^41^. Vertical tidal oscillations of the groundwater table in the beach acted like a piston pump, drawing warm, oxygen-saturated air into the sand, and moist, carbon-dioxide enriched air from deeper layers to the surface, keeping oxygen at >50% air saturation throughout the upper 50 cm of the beach^10^ (Fig. S2c). Thus, microbial decomposition of buried SOA hydrocarbons could proceed aerobically^15^, outpacing anaerobic petroleum hydrocarbon degradation by far^42^. Homogenous color change across the cross section of the sSOAs and similar carbon content in the center (8.9%, 1 SD = 0.9%) and outer shell (9.3%, 1 SD = 0.3%) of SOAs collected at the study site support that aerobic microbial decomposition progressed throughout the permeable SOAs. Furthermore, tidal beach ventilation maintained temperatures exceeding 20 °C in the upper 50 cm of the beach from April to October^10^, accelerating the biodegradation of petroleum hydrocarbons^43,44^. Although temperature in the Florida beach sand increases toward the surface except during winter^10^, decay rates of *n*-alkane and PAHs in general increased with sediment depth, and the decay of the *n*-alkanes reached maximum rates at 40 cm depth. This suggests that moisture may have been a limiting factor for biodegradation in the surface layer of the beach, where high Florida solar radiation and temperatures enhanced evaporation. A peak in decay rate at 20 cm sediment depth found for the majority of PAHs (Fig. 4d) suggests that PAH degradation was influenced more strongly by temperature than *n*-alkanes degradation. The enhancing effect of warm temperatures on PAH degradation thus may have offset the inhibiting effect of lower moisture content in the upper sediment layers. A moisture content of 1.7% is sufficient for sedimentary oil biodegradation^45^, and air pumped upwards from deep, tidally wetted sediment horizons to the beach surface likely provided the moisture satisfying these needs in the subsurface layers^10^. Moisture produces a thin fluid film on the sand grains, through which molecular diffusion can transport dissolved nutrients to the microbes colonizing sediment and oil particles. Biodegradation of oil can proceed at C/N/P ratios of 100/1.7/0.2^43^, and effective biodegradation of PAHs maybe possible at a C/N/P ratio of 100/1.3/0.05^46^. MC252-SOAs collected on Louisiana beaches had a C/N/P ratio of 100/1/0.2^47^. Assuming that the sSOAs and sSRBs had similar C/N/P ratios, their biodegradation was not blocked by a lack of nutrients as supported by the observed decay rates. Nutrient input as could be caused by runoff after rain events or storm surges that inundate the beach can enhance the oil degradation rate^41,48^. An experiment assessing the degradation of light crude oil in the intertidal zone of a Delaware Bay beach and a subsequent modeling study suggested that the oil biodegradation rate could be doubled by addition of nutrients^49,50^. Likewise, laboratory experiments by Horel *et al*.^51^ revealed that the presence of degrading saltmarsh plant or fish tissue could enhance biodegradation of MC252 oil in water-saturated sands by 25 to 123%, respectively. Our experiment was designed to integrate such nutrient-related enhancement effects through the incubation of sSOAs in the beach over a 3-year period. During this time frame, rain storms and deposition of organic material onto the beach presumably caused temporary increases of nutrient availability for the oil biodegradation process, which is partly reflected by the deviations of the calculated decay rates from the calculated decay curves.

The *n*-alkane and PAH half-lives found in this *in-situ* study are within the range of published half-lives reported for similar shore and coastal environments (Table 1). However, Elango *et al*.^52^ calculated half-lives of 70d for *n*-alkanes and 83d for PAHs in DWH-SRBs from Louisiana’s Fourchon Beach and Elmer’s Island, which are very similar to the 53d to 67d half-lives for PAHs found in the Pensacola Beach intertidal zone by Snyder *et al*.^53^. These half-lives along with other half-lives of DWH-related SOAs reported in Table 1 are shorter than those we found for the sSOAs, likely because the observation intervals were shorter and environmental settings controlling SOA decay (moisture, nutrients, mechanical stress) in these studies differed from those in the dry Pensacola beach sand. In the determination of the average decay rate of a hydrocarbon mixture, a shorter observation interval gives more weight to the more degradable compounds that initially are more abundant. Inclusion of the heavier *n*-alkanes (C_32_-C_40_) in our calculations therefore may have resulted in slower average decay rates. Furthermore, the location within the beach environment may be critical for the SOA degradation rate. A comparison of our results obtained in the supralittoral dry beach with half-life times determined for light crude oil (Bonny Light) degradation in the periodically submersed intertidal beach suggests that petroleum hydrocarbon decomposition in the supralittoral zone may be about one order of magnitude slower than in the intertidal zone (halflives alkanes: 27d, aromatics 33d^50,54^), emphasizing the role of the periodical inundations for the supply of water and nutrients to the oil-degrading microbial community. This hypothesis is supported by observations of relatively rapid MC252 SOA degradation in the intertidal zone of Pensacola Beach^10^ (Table 1).

**Table 1:** Comparison of half-lives of petroleum hydrocarbons reported for coastal marine environments (half-life = LN(2)/decay rate constant).

Source	Type of Oil Deposit	Half Life Alk (d)	Half Life PAH (d)	Location or origin	Event	Reference
varied	crude oil	<25–383	38–898	model	modeling decay	Howard *et al*. 2005
varied	—	—	61–2190	literature value	synthesis	Shiu *et al*. 2006
**buried supratidal MC252**	**SOA**	**285 (100–568)**	**352 (94–836)**	**Pensacola Beach, FL**	**DWH blowout**	**this study**
buried intertidal MC252	weathered oil	53	58	Pensacola Beach, FL	DWH blowout	Huettel *et al*. 2018
surface supratidal MC252	SRB	70	83	Caminada Headlands, LA	DWH blowout	Elango *et al*. 2014
surface shelf MC252	crude	—	53–67	Florida shelf	DWH blowout	Snyder *et al*. 2014
shelf MC252	SOM	6	—	Louisiana shelf plume	DWH blowout	Hazen *et al*. 2010
intertidal re-exposed MC252	SRB	—	1733*	Gulf Shores, AL	DWH blowout	John *et al*. 2016
surface marsh MC252	weathered oil	63*	53*	Barataria Bay, LA	DWH blowout	Mahmoudi *et al*. 2013
surface estuary MC252	weathered oil	178*	—	Louisiana estuaries	DWH blowout	Turner *et al*. 2014
surface sediment Bonny	crude oil	27	33	Delaware shoreline	experiment	Venosa *et al*. 1997
surface intertidal Prestige	light crude oil	92–241	64–317	Galicia, Spain	experiment	Jimenez *et al*. 2006
surface sediment submerged	weathered #4 crude	62*	112	Galicia, Spain	Aegean Oil spill	Pastor *et al*. 2001
buried intertidal	Brent crude oil	—	161	Sequim Bay, Washington	experiment	Anderson *et al*. 1983
intertidal surface	weathered oil	—	61–487	Prince William Sound, AK	Exxon Valdez spill	Boehm *et al*. 1995
buried intertidal Exxon	weathered crude	—	61–487	Prince William Sound, AK	Exxon Valdez spill	Wolfe *et al*. 1994
intertidal surface Exxon	weathered oil	—	866–1155	Prince William Sound, AK	Exxon Valdez spill	Peterson *et al*. 2005
surface water light crude	weathered oil	—	73	Breton coast	Amoco Cadiz spill	Berne *et al*. 1980
stranded surface water	weathered tanker oil	70	88	Korean coastlines	Hebei Spirit oil spill	Yim *et al*. 2011
intertidal	crude tanker oil	—	43	Puget Sound	ARCO Ancorage spill	Miller 1989
beach surface	weathered tanker oil	—	123*	Clifton Beach, Pakistan	Tasman Spirit spill	Munshi *et al*. 2011

*Values calculated from publication data (not reported by actual publications).

**Figure 6:**
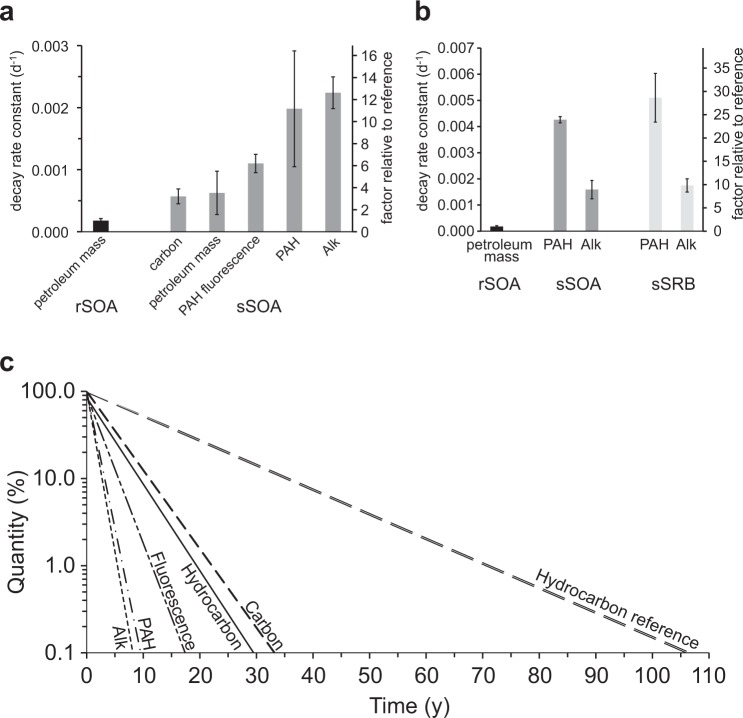
Summary of decay rates and projections. (**a**) Decay rates of rSOAs and sSOAs. The factor relative to reference relates the decay rates to the mass loss rate of the reference rSOA kept in the dark at constant temperature. Error bars depict standard error. (**b**) Decay rates of rSOAs, sSOAs and sSRBs during the first 9 months for which sSRB data are available. (**c**) Projections of petroleum hydrocarbon decomposition in sSOAs based on the decay rate constants determined in our study. Lines depict percent quantity remaining versus time. sSOA carbon content decreases slower than sSOA petroleum hydrocarbon mass (which includes oxyhydrocarbons) because small amounts of inorganic carbon may be contained in the sSOAs. Fluorescence decays slower than the GC/MS amenable PAHs as some oxidized PAHs retain fluorescence. Petroleum hydrocarbon mass in the reference rSOAs kept in the dark at constant temperature decays slower because the rSOAs were not exposed to the complex microbial community in the beach sand and the sedimentary environmental settings.

Analysis of MC252-SRBs collected from Florida, Alabama, Mississippi, and Louisiana beaches revealed a consistent chemical composition^55^, suggesting that the results found at our Pensacola Beach study site may also apply to other Gulf sandy beaches. Assuming that the exponential first order decay continues at the rates observed in the 3-year *in-situ* experiment (Fig. 6a), these rates can be used to estimate the minimum lifetime of golf ball-size SOAs buried in sandy Gulf beaches (Fig. 6c). Lifetime here is defined as the period from the deposition on the beach to the time point when the material has decreased to 0.1% of its initial amount.

The decline of sSOA hydrocarbon mass at rate constants of 0.00063d^−1^ and carbon content at 0.00057d^−1^ puts the minimum lifetime of golf-ball-size MC252-SOAs buried in Florida beach sands at 28 to 32 years. The GC/MS amenable, non-oxidized sSOA hydrocarbon compounds, degraded about 3-times faster (Alk: 0.0022d^−1^, PAH: 0.0020d^−1^, depleted after 8 to 9 years), implying that the decomposition of the oxyhydrocarbons resulting from the oxidation of the *n*-alkanes and PAHs^56^ was substantially slower (depleted after ~30 years), as supported by the findings of Aeppli *et al*.^18,57^. At the beach surface, photolysis^9,38^ enhanced the decomposition of PAHs by about 16% (Fig. 6b). This had a limited impact on the degradation of sSRBs relative to buried sSOAs because *n*-alkanes comprised the majority of petroleum hydrocarbons in the agglomerates (MC252 source oil: 74% Alks, 16% PAHs^20^, stranded oil mousse: ~22% PAH + alkylated PAHs^33^). Alkanes were not affected by photodegradation and decomposed about 10% faster within the sediment. The enhancement of biodegradation by the beach sedimentary environment (warm temperature, moisture, nutrient supply) becomes apparent when comparing the *in-situ* decomposition of the agglomerates in the beach to the degradation of the reference rSOAs that were kept in the lab under dark and constant temperature conditions. After 7.4 years of lab incubation, the rSOA petroleum hydrocarbon mass had decreased by 38% (1 SD = 5%), and their decay (0.0002d^−1^) thus was 3-times slower than that of the buried sSOAs (0.0006d^−1^). Extrapolating this trend suggests that the reference rSOAs would persist for at least 105 years. Because the aliquots of rSOA material kept in the lab were smaller than the sSOAs buried in the beach (1.4 g vs. 33.7 g), their surface to volume ratio was about 30-times larger than that of the sSOAs. This enhanced oxygen availability to the microbial community in the rSOAs compared sSOAs. The microbial decay rates of the rSOAs therefore should be considered conservative when comparing with sSOA decay rates.

Although our results indicate that the majority of petroleum hydrocarbons in the golf-ball-sized MC252 sediment-oil-agglomerates may decompose within three decades, a small portion of their crude oil compounds may persist over longer periods. The exponential decay functions calculated for sSOA hydrocarbon mass and carbon content may include slow-degrading compounds (e.g. large polar resins and asphaltenes^58,59^) that have a minimal impact on overall decay because they represent a minor fraction (<10%) of the MC252-oil components^20^. Aeppli *et al*.^18^ concluded from analysis of SRBs collected between 2011 and 2017 along Gulf shores that the persistence of oxyhydrocarbons limited the overall crude oil hydrocarbon depletion in these MC252 agglomerates to 42% (1 SD = 12%) after 7 years, which equates to approximately half of the 76 to 83% depletion estimated by our *in-situ* experiment for that time period. MC252-SRBs continue to wash up onto Gulf beaches to the present day, and a possible explanation for this discrepancy could be the formation of SRBs from oil that was temporarily embedded in sublittoral sediments^60^ where protection from oxygen substantially reduced degradation rates and preserved some of the *n*-alkanes and PAHs as also found after the Falmouth oil spill^42,61,62^.

The relatively large variability of published decay rates for crude oil compounds and SRBs (Table 1) may partly be explained by the central role of surface to volume ratio for crude oil degradation in the environment. Petroleum hydrocarbon degradation is largely a surface phenomenon^63,64^, and therefore a function of size, shape and porosity of the SOAs. The spherical SOAs used in our experiment had a ~40-times smaller surface per volume ratio compared to millimeter-size oil particles, reducing the exposure of their hydrocarbons to biodegradation, similar to what was observed in suspended oil droplets of different size^65^. Millimeter-size range oil particles and the oil film adhering to sand grains in the upper 70 cm of the Pensacola beach sand degraded to background hydrocarbon levels within one year, while the larger buried SOAs persisted^10^. Our current study estimates a ~30-year period for the decomposition of golf-ball-sized SOAs, underlining the role of the surface to volume ratio. Nevertheless, our results also show that the SOAs are porous and permeable, which greatly extends the surface area available for aerobic microbial attack, supporting relatively rapid decomposition of the larger agglomerates.

The comparison of the decay rates of the agglomerates in the beach and of those kept in the lab under dark and constant temperature reveals the enhancement of the oil decomposition by the complex interacting microbial community in the beach and the environmental factors influencing biodegradation. Regular exchange of the air in the pore space of the sand caused by the tidal vertical oscillation of the groundwater level in the beach^10^ and the permeability of the agglomerates to gas facilitate aerobic microbial degradation of buried sand-oil-agglomerates. This may explain the faster decomposition of the SOAs buried in the permeable beach in comparison to oil degradation in anoxic settings or in fine-grained sediments with low permeability^23,42,66,67^. In the intertidal zone of sandy beaches, the increased moisture and nutrient supply caused by regular inundation can further enhance buried SOA degradation by an order of magnitude^50,54^. The confined ~3 decades degradation period for this most common SOA size (<10 cm) used in our study can explain why sand-oil-agglomerates, produced from natural and anthropogenic oil sources and continuously washing onto Gulf of Mexico shores, do not accumulate in the beach, emphasizing the role of the sandy beach as an aerobic biocatalytical reactor. Sediment-oil-agglomerates with 2500-times larger volumes than those studied here were observed after the Deepwater Horizon spill at the study site (Fig. S7), underlining the need for assessing agglomerate dimensions and sediment permeability when estimating beach recovery time after oil spills^68–83^.

## Supplementary information

Supporting Information

## Data Availability

Data of this study are available through the supplemental information and the GoMRI GRIIDC data server at https://data.gulfresearchinitiative.org/.
