# Supplementary material for: Decomposition of sediment-oil-agglomerates in a Gulf of Mexico sandy beach
Source: Sci Rep. 2019 Jul 11;9:10071. doi: 10.1038/s41598-019-46301-w (PMC6624294; doi:10.1038/s41598-019-46301-w)
Supplement: Supplementary file 1 — Supporting Information [file 41598_2019_46301_MOESM1_ESM.pdf]

Supporting Information

Decomposition of sediment-oil-agglomerates in a Gulf of Mexico sandy beach

Ioana Bociu, Boryoung Shin, Wm. Brian Wells, Joel E. Kostka, Konstantinos T. Konstantinidis, Markus Huettel*

*Corresponding author: Markus Huettel, mhuettel@fsu.edu, (850) 645-1394

	Pages
Supporting text for methods section	S2 – S4
Supporting Figures S1-S7	S5 – S8
Supporting Tables S1–S6	S9 – S13

Supporting text

Fluorescence measurement method. The fluorescence of the surface of each sSOA was measured with a fluorometer (Waltz™) that allowed attachment of a fiber optic cable for solid-surface fluorescence determination. For the measurement, the sSOA was placed in a light-tight jar with opaque black walls. A hole in the lid of the jar allowed insertion of the fiber-optic cable. A black velvet sheet covering the jar and inserted fiber-optic cable blocked any light penetration into the jar. A collar attached to the end of the fiber-optic cable ensured that all fluorescence measurements used the same distance between the cable tip and the sSOA. A reference sample of the homogenized material used to produce the sSOAs and kept frozen between measurements was measured before and after each sample measurement. A calibration line generated by measuring the fluorescence of known mixtures of the reference material and clean sand (0-100% by weight) was produced, allowing expression of the results as percent of the initial sSOA material fluorescence remaining at the sampling time of the sSOA.

sSOA petroleum hydrocarbon extraction. To the bottom of each of 6 extraction flow-through cells, 3 g of pre-combusted sodium sulfate (450°C for 4.5 hours) was added for moisture removal. Then, 12 g of pre-combusted silica gel (250°C for 4.5 hours) was filled in each column for removal of organic contaminants (in-cell clean-up). The homogenized sSOA aliquot, mixed with 1 g of sodium sulfate, was added to the top of the silica gel. Finally, another 3 g of pre-combusted sodium sulfate were applied to cover the sSOA mixture. Each filled flow-through cell was spiked with 160 µL (1000 mg kg^{-1}) of

surrogate standard. Then mass spectrometer grade methylene chloride (DCM) was cycled through the flow-through cells twice at a pressure of 140 bar and a temperature of 100°C. The solvent was heated for 1 minute, then held for 5 minutes in the cell, and afterwards discharged from the cell over a 3-minute time period. The cells then were flushed with solvent for 1 minute, followed by flushing with N_2 gas for 3 minutes.

GC/MS Analysis. The injection volume into the GC/MS system was set to 1µL. The multimode inlet temperature was kept at 280°C on pulsed splitless mode, with a 1.2 mL min^{-1} main column gas flow, and 2 mL min^{-1} total gas flow when including the backflush column. The GC/MS transfer line heater, source and quadrupoles were kept at a constant 300°C, 300°C and 150°C, respectively. Collision cell EPC gas flow was 1.5 mL min^{-1} of N_2 and 2.25 mL min^{-1} of He. For saturated hydrocarbon analysis, the oven ramp program started at 50°C with a 5 minute hold, continued to 320°C at 30°C min^{-1} with a 8 minute hold, followed by a 10°C min^{-1} ramp rate to 325°C with a 20 minute hold, and ended with a 325°C post run for 4 minutes. The oven ramp program for aromatic hydrocarbons started at 70°C with an increase to 140°C at 20°C min^{-1} with a 4 minute hold. This was followed by a 4°C min^{-1} ramp to 205°C, continuing at 25°C min^{-1} from 205°C to 300°C, and at 20°C min^{-1} to 325°C with a 22 minute hold. The program ended with a 325°C post run for 4 minutes. The electron ionization system of the mass spectrometer used an ionization energy of 70 eV delta EMV, and a peak width of 0.7 m/z. Multiple-reaction-monitoring (MRM) methods were utilized for the quantitative evaluation of the mass spectrometer data of saturated and aromatic hydrocarbons.

Oxygen measurements in sSOAs. Two methods were used to install the oxygen fiber sensor in the center of the sSOAs. 1) Two sSOAs (not previously buried or aged) were placed on weighing dishes. A stainless steel needle then was used to produce a narrow vertical hole (1 mm diameter) from the surface to the center of the sSOAs using a micromanipulator. Afterwards, a calibrated oxygen fiber optode (Pyroscience™, 430 µm fiber diameter) was vertically inserted into the hole with the micromanipulator, such that the sensing tip of the sensor was positioned in the center of the sSOAs. The sSOA was slightly compressed to seal the fiber in the sSOA and the location where the fiber entered the sSOA was sealed with SOA material and a drop of mineral oil. 2) A sSOA (not previously buried or aged), contained in a stainless steel teaball, was cut in half where the halves of the teaball meet, and teaball/sSOA halves were separated such that their cross-sections were horizontal. An 8 mm long piece of silicone tubing (0.8 mm inner diameter, 2.3 mm outer diameter) was horizontally embedded in the center of the cross section of one of the two sSOA half-spheres. An oxygen fiber optode (Pyroscience™, 430 µm fiber diameter) was inserted with a micromanipulator into the tubing such that the sensing tip was located in the center. A permeable cotton plug closed in the open end of the tubing. The second half of the sSOA then was pressed firmly onto the first sSOA half. Because the sSOA material was relatively soft (similar consistency as play-doh™), this procedure sealed the fiber and silicone tubing in the sSOA. The location, where the fiber entered the sSOA additionally was sealed with a drop of mineral oil. The oxygen concentrations in the center of the three sSOAs were measured with a Pyroscience™ FireStingO2 oxygen meter connected to a HP-laptop computer.

Supporting Figures

Figure S1. Location of the study site (yellow circle) at Pensacola Beach (30° 20' 55.8" N, 87° 02' 52.2" W), and the area affected by the Deepwater Horizon (DWH) surface oil slick (black area).

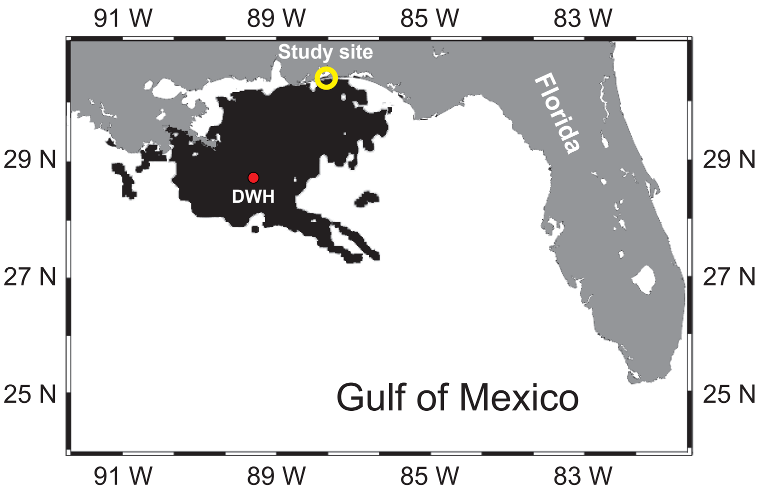

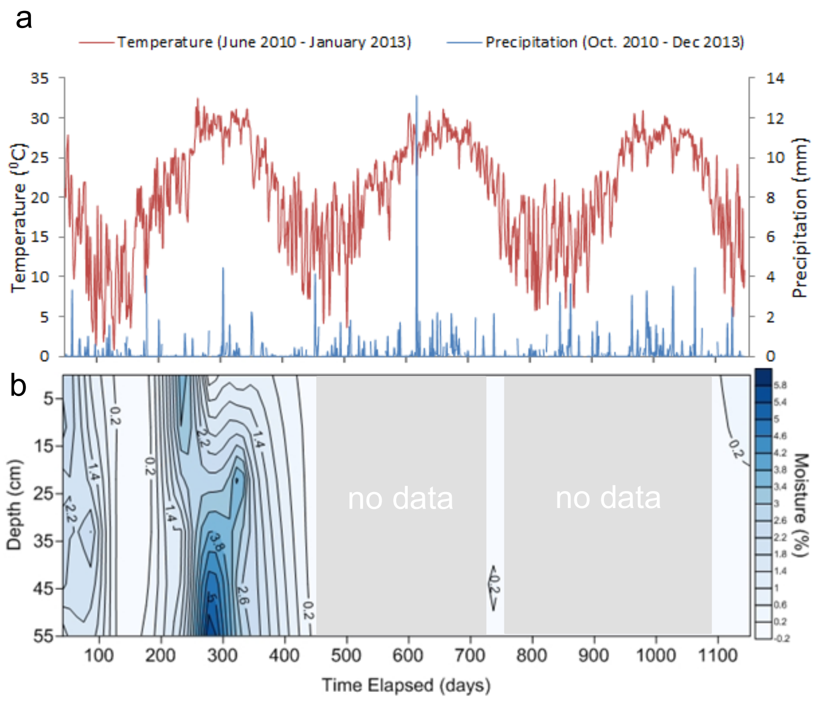

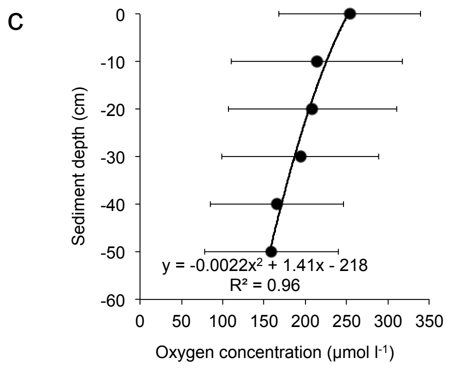

Figure S2. a: Precipitation and temperature at Pensacola Beach over the three-year experimental time period. Data are from the Florida Climate Center Downloadable Data Tool station PENSACOLA RGNL AP. b: Moisture content of sediment surrounding oil-sand agglomerates over depth, with samples for moisture analysis being removed at the same time as sSOAs. c: Sediment oxygen profile measured at the study site on 26 October 2012

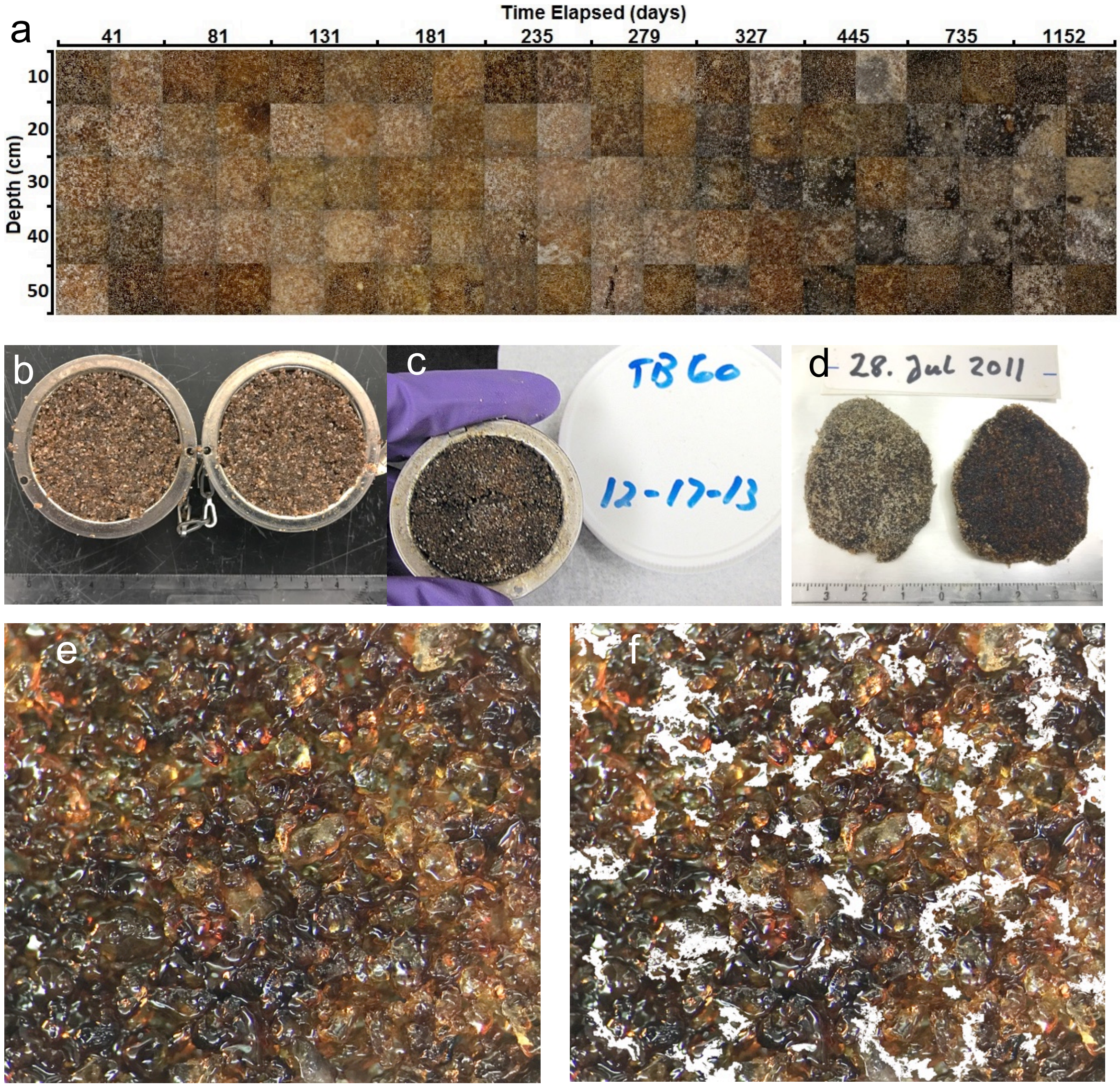

Figure S3. a: Coloration of sSOAs retrieved from the different sediment depth during the experiment. For each sampling day indicated on the X-axis, two photographs are shown, one for each of the two sSOAs buried at the depth indicated on the Y-axis. b: Cross sections of an sSOA at the beginning (10th Oct. 2010) and c: at the end of the experiment (17th Dec. 2013). d: Surface (left) and cross section (right) of an SOA collected at Pensacola Beach on 28th July 2011. e: Close-up of the cross-section shown in D. The dark color of the oiled sand results from a thin oil film coating each sand grain while larger pore spaces remain open. f: Open pore space deeper than approximately 1 grain diameter (400 µm) seen in E were marked white. Approximately 30% of the SOA volume is porespace filled with air (calculated from SOA volume and the weights of the sand, water and oil it contained).

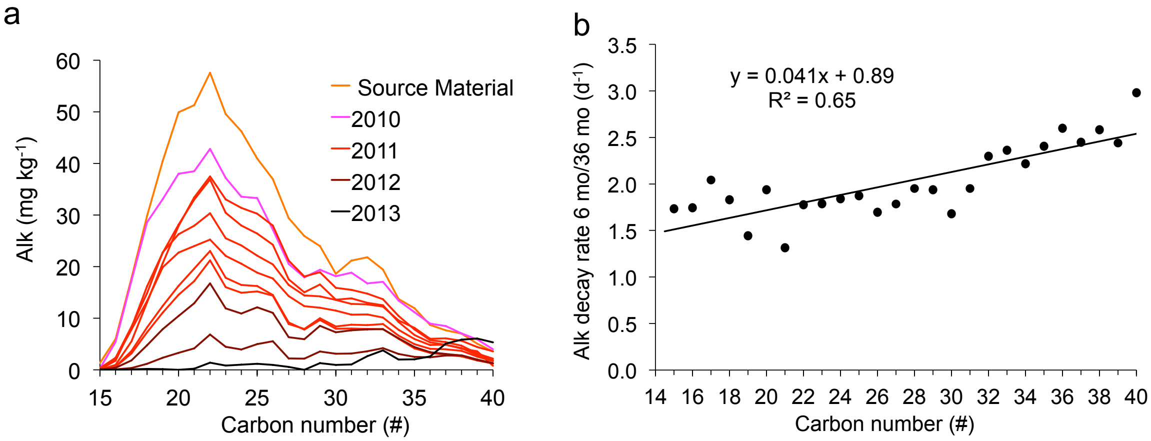

Figure S4. a: Concentrations of C15-C40 alkanes during the three years of the experiment. The concentration increases for C > 31 in 2013 are an artifact likely caused by column bleeding and not present in all samples (e.g. Fig. S6) b: Figure S6. Increase of the 0.5 year/3 year decay rate constants ratio by carbon number.

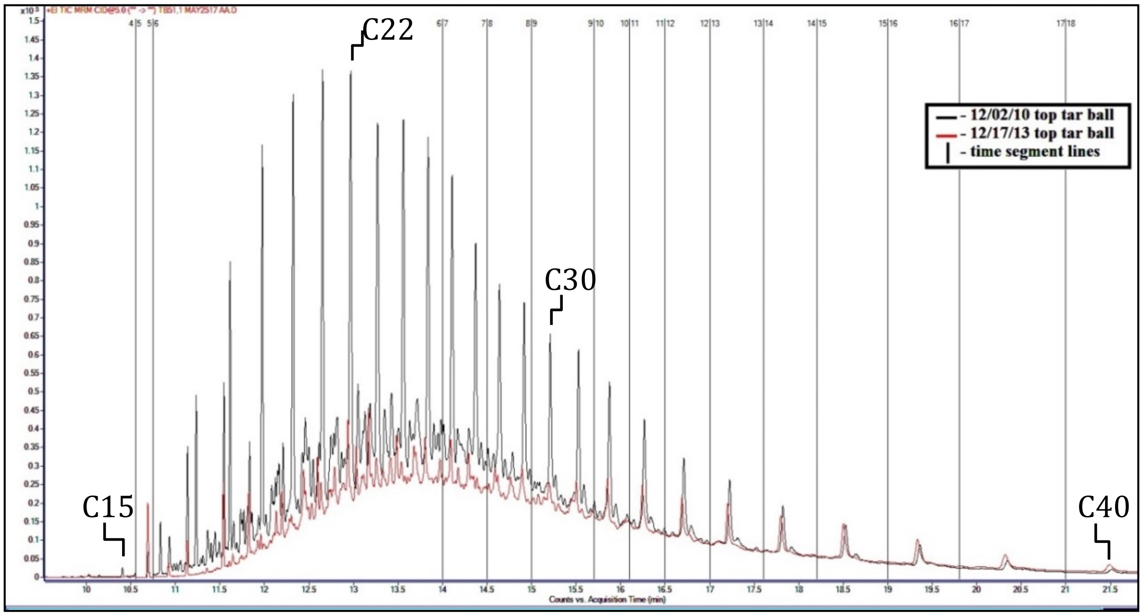

Figure S5: Chromatograph of *n*-alkanes, with carbon numbers identified (C15, C22, C30, C40). The column used is preferred for PAH's, which shows highest signal values in middle retention times. This makes the highest signal for saturated alkanes show in the middle time segment as well. The black line represents the 10 cm depth sSOA of the December 2010 array, and the red line is the 10 cm depth sSOA of the December 2013 array. The heavier saturated alkanes are less susceptible to degradation.

Figure S6. PAH decay rates versus number of C_6 rings. Black circles: Decay rates over the first half year. Gray circles: Decay rates over two years.

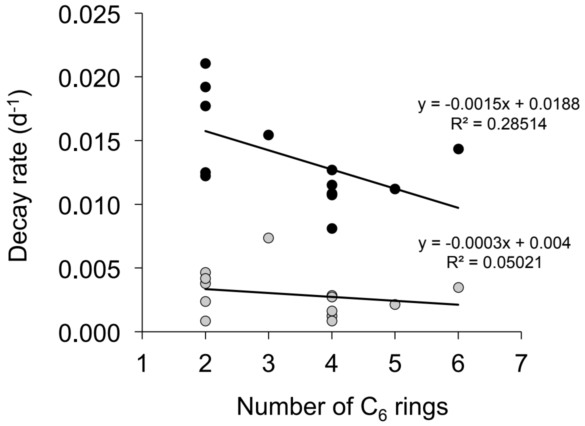

Figure S7. Partly exposed large SOA buried in Pensacola Beach sands after the Deepwater Horizon accident. This SOA was approximately 82 cm long, 42 cm wide and 25 cm thick, with a volume of 87700 cm^3, or 2500 times larger than our experimental sSOAs (Photo Huettel, 30th June 2010)

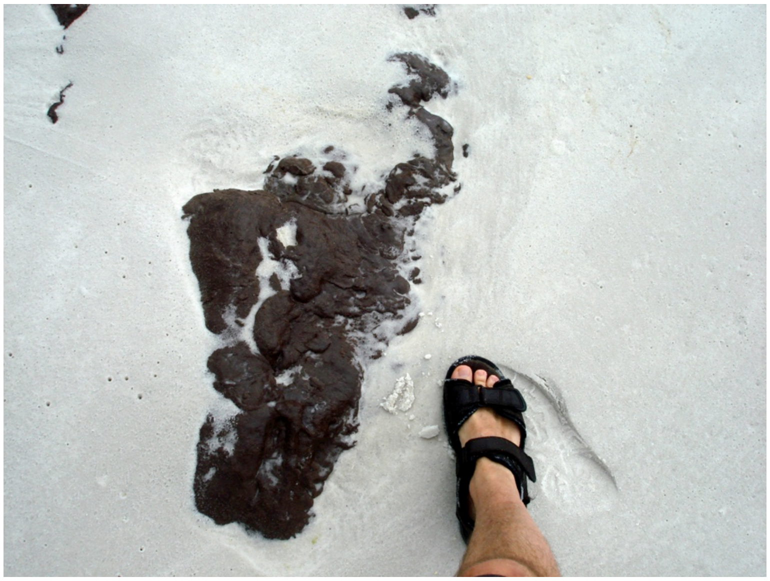

Supporting Tables

Table S1: Alkane compounds monitored in order of elution with quantifying and qualifying ions and the internal standard used for each compound.

Alkane Compound Elution Order										
				Precursor Ion	Product Ion			Product Ion		
	Compound	Type	ISTD	Quantitative	Qual	CE (eV)	Dwell	Qual	CE(eV)	Dwell
1-6	C10-C15	target	ISTD-1	85.1	71.1	5	50	57.1	5	50
7	d24-hexadecane	ISTD-1		98.2	66.1	5	50	82.2	5	50
8	C16	target	ISTD-1	85.1	71.1	5	50	57.1	5	50
9	pristane	Surrogate	ISTD-1	85.1	71.1	5	50	57.1	5	50
10	C17	target	ISTD-1	85.1	71.1	5	50	57.1	5	50
11	phytane	Surrogate	ISTD-1	85.1	71.1	5	50	57.1	5	50
12-34	C18-C40	target	ISTD-1	85.1	71.1	5	50	57.1	5	50

Table S2: PAH compound elution order with quantifying and qualifying ions and internal standards. Collision energy (CE) is in eV and dwell time (Dwell) is in seconds.

PAH Compound Elution Order (EPA & IARC)										
				Precursor	Product Ion			Product Ion		
	Compound	Type	ISTD	Quantitative	Qual	CE (eV)	Dwell	Qual	CE(eV)	Dwell
1	DB-naphthalene	ISTD-1	ISTD-1	136.1	108	25	10			
2	Naphthalene	target	ISTD-1	128	127	20	10	102	20	10
3	2-Fiuorobiphenyl	Surrogate	ISTD-1	172	171	15	10	170	30	10
4	Biphenyl	target	ISTD-1	154	153	15	10	152	30	10.8
5	Acenaphthylene	target	ISTD-1	152	151	20	10	150	35	12.8
6	2-Bromonaphthalene	Surrogate	ISTD-1	205.9	127	20	19.4	151	35	20.9
7	Acenaphthene	target	ISTD-1	153	152	20	10	164	40	14.2
8	Fluorene	target	ISTD-1	166	165	20	10	152	25	10
9	Dibenzothiophene	target	ISTD-1	184	139	35	10.3	187	5	19.4
10	DlO-phenanthrene	ISTD-2		188.1	184	35	10			
11	Phenanthrene	target	ISTD-2	178	176	35	10	152	25	10
12	DlO-anthracene	Surrogate	ISTD-2	188.1	187	35	10	187	5	19.4
13	Anthracene	target	ISTD-2	178	176	35	10	152	25	10
14	Ortho-terphenyl	Surrogate	ISTD-2	230	215	20	10	229	15	10
15	Fluoranthene	target	ISTD-2	202	200	40	10	201	25	10
16	DlO-pyrene	Surrogate	ISTD-3	212.1	208	45	10	210	30	10
17	Pyrene	target	ISTD-3	202	200	40	10	201	25	10
18	Benzo(c)phenanthrene	target	ISTD-3	228	226	40	10	227	20	10
19	Benz(a)anthracene	target	ISTD-3	228	226	40	10	227	20	10
20	D12-chrysene	ISTD-3		240.1	236	40	10			
21	Chrysene	target	ISTD-3	228	226	35	10	227	20	10
22	Benzo(b)fluoranthene	target	ISTD-4	252	250	40	10	126	50	69.3
23	Benzo(k)fluoranthene	target	ISTD-4	252	250	40	10	253	50	69.3
24	7,12-dimethylbenz(a)	target	ISTD-3	256	241	45	10	239	45	10
25	Benzo(j)fluoranthene	target	ISTD-4	252	250	40	10	126	50	69.3
26	Benzo(e)pyrene	target	ISTD-4	252	250	40	10	251	40	10
27	Benzo(a)pyrene	target	ISTD-4	252	250	20	13.6	251	20	10.6
28	D12-perylene	ISTD-4		264.1	260	40	10.6			
29	Perylene	target	ISTD-4	252	250	40	10	251	20	10
30	3-Methylcholanthrene	target	ISTD-4	268	252	40	10	253	20	10
31	ndeno(l,2,3-c,d)pyrene	target	ISTD-4	276	274	45	10	275	30	10
32	Dibenz(a,h)anthracene	target	ISTD-4	278	276	40	10	277	20	14.8
33	Benzo(g,h,i)perylene	target	ISTD-4	276	274	50	17.5	138	50	17.5
34	Dibenzo(a,I)pyrene	target	ISTD-4	302	150	40	10	300	40	56.1
35	Dibenzo(a,i)pyrene	target	ISTD-4	302	151	40	10	300	40	56.1
36	Dibenzo(a,h)pyrene	target	ISTD-4	302	151	40	10	300	40	56.1

Table S3. Concentrations of *n*-alkanes measured during the experiment. The sample for day 279, 40 cm depth was lost.

Day after start	Depth (cm)	C15	C16	C17	C18	C19	C20	C21	C22	C23	C24	C25	C26	C27	C28	C29	C30	C31	C32	C33	C34	C35	C36	C37	C38	C39	C40	Sum
		($mg\ kg^{-1}$)																										
0	0	1	6	18	30	40	50	51	58	50	46	41	37	29	26	24	19	21	22	19	14	12	9	8	7	5	4	646
41	10	0	4	12	22	27	34	34	38	35	31	30	25	19	17	17	16	17	14	13	11	8	7	6	5	4	3	447
41	20	0	4	14	26	31	37	39	44	39	34	34	28	21	17	19	19	19	17	16	13	11	9	8	7	5	3	515
41	30	0	7	22	35	39	44	44	48	41	37	37	30	23	20	21	20	21	19	20	16	13	10	10	8	7	5	597
41	40	0	7	20	32	36	40	40	43	37	34	34	28	21	18	20	19	20	18	19	15	12	10	9	8	7	5	552
41	50	0	5	18	29	32	35	36	41	34	32	31	25	19	17	19	17	18	17	18	14	12	9	8	7	6	4	503
89	10	0	3	9	16	22	26	26	28	25	23	21	20	16	14	14	13	12	12	12	9	8	6	6	4	4	2	350
89	20	0	2	8	14	20	23	25	25	23	21	19	19	15	13	12	12	12	11	10	8	7	5	5	4	4	0	317
89	30	0	2	7	13	20	23	24	26	22	21	19	18	14	12	12	11	10	11	10	8	4	5	5	4	3	1	305
89	40	0	2	7	12	18	21	22	23	21	19	17	16	13	11	11	10	9	9	9	6	5	4	4	4	3	0	276
89	50	0	2	7	13	19	22	23	25	21	20	18	17	13	12	11	10	11	10	10	8	6	4	5	4	3	0	293
131	10	0	2	8	15	24	28	30	32	27	26	24	22	17	15	15	15	13	12	12	10	9	6	6	6	5	4	386
131	20	0	3	9	17	25	28	29	30	27	26	24	22	18	16	16	14	15	15	13	11	9	7	7	6	5	4	396
131	30	0	2	8	14	22	26	27	31	25	23	22	20	16	14	14	13	13	12	12	9	8	6	6	6	4	4	361
131	40	0	2	8	14	22	25	27	29	24	23	21	20	16	14	13	13	11	12	12	9	8	6	6	5	4	3	347
131	50	0	2	8	13	21	24	26	29	24	22	20	18	15	13	13	12	11	12	12	9	7	5	6	5	4	3	336
181	10	0	1	7	16	23	29	34	39	30	28	27	25	17	14	17	14	14	13	12	9	7	5	4	3	2	1	391
181	20	0	1	6	13	18	23	27	31	24	22	22	20	13	12	14	11	12	11	11	9	6	5	5	4	3	2	325
181	30	0	0	1	4	8	11	13	19	13	13	14	13	8	8	10	9	9	9	9	7	6	4	4	3	2	2	199
181	40	0	0	0	0	0	0	1	3	1	0	2	3	0	0	1	1	1	2	3	1	1	2	3	4	3	2	36
181	50	0	0	1	3	6	9	11	15	11	11	12	11	6	6	8	8	8	8	9	7	5	4	4	4	3	2	173
235	10	0	3	11	21	28	34	41	44	37	34	31	29	22	19	20	16	17	15	14	11	8	6	6	5	3	2	475
235	20	0	2	9	17	24	30	34	37	32	29	28	25	19	16	17	14	15	14	13	10	7	6	5	5	3	2	414
235	30	0	2	7	15	21	26	29	33	28	26	24	22	17	14	15	13	13	12	12	9	6	5	4	4	3	2	360
235	40	0	1	7	16	23	29	34	39	30	28	27	25	17	14	17	14	14	13	12	9	7	5	4	3	2	1	391
235	50	0	1	6	13	18	23	27	31	24	22	22	20	13	12	14	11	12	11	11	9	6	5	5	4	3	2	325
279	10	0	1	6	14	23	31	37	40	36	35	34	31	24	20	21	18	17	16	15	11	8	6	6	5	3	2	462
279	20	0	1	6	13	20	27	33	38	33	32	30	28	22	18	19	16	16	15	14	11	8	6	6	5	3	2	423
279	30	0	1	4	11	17	24	30	35	31	30	28	26	20	17	17	15	14	14	12	10	8	5	5	4	3	2	385
279	40	n/a	n/a	n/a	n/a	n/a	n/a	n/a	n/a	n/a	n/a	n/a	n/a	n/a	n/a	n/a	n/a	n/a	n/a	n/a	n/a	n/a	n/a	n/a	n/a	n/a	n/a	n/a
279	50	0	1	6	14	22	29	33	37	32	29	28	26	20	17	18	15	15	14	13	10	7	6	5	5	3	2	408
327	10	0	1	6	12	17	22	27	30	23	21	20	18	12	10	12	10	10	9	9	7	4	3	3	3	2	1	293
327	20	0	1	4	9	14	18	22	25	20	18	18	16	10	9	10	8	9	8	8	6	4	3	3	3	2	1	248
327	30	0	0	2	4	6	8	9	13	9	8	9	8	4	4	6	4	5	6	7	5	4	3	3	2	2	1	131
327	40	0	1	5	11	16	22	26	30	24	22	22	19	13	11	12	10	10	9	9	6	4	3	3	2	2	1	293
327	50	0	0	3	6	9	12	14	18	13	12	13	11	7	6	8	7	7	7	7	6	4	3	3	3	2	1	183
445	10	0	1	2	4	6	7	9	13	8	7	9	9	4	4	8	7	8	8	9	7	4	4	3	3	2	1	146
445	20	0	0	2	6	10	13	17	19	15	14	14	13	8	7	9	8	8	8	8	7	5	3	3	3	2	1	203
445	30	0	0	1	1	2	2	3	7	4	3	6	5	1	2	4	3	4	5	5	4	4	3	3	2	2	1	77
445	40	0	0	2	7	12	16	20	24	18	17	17	15	10	9	11	10	10	9	9	7	5	4	3	3	2	1	241
445	50	0	0	2	6	9	13	16	21	15	14	15	13	8	8	10	9	9	9	8	6	5	4	3	3	2	1	209
735	10	0	0	1	3	7	10	11	16	11	11	12	11	7	6	9	7	8	8	9	7	5	4	4	3	2	2	174
735	20	0	0	0	0	0	0	0	1	1	0	1	2	0	0	1	1	1	1	1	0	0	1	2	3	2	1	22
735	30	0	0	1	2	4	7	8	12	8	8	9	9	4	4	6	5	6	6	8	6	5	4	4	4	3	2	134
735	40	0	0	0	0	0	0	1	3	1	1	2	3	0	0	1	1	1	2	2	2	2	2	2	2	1	1	29
735	50	0	0	0	0	0	0	1	2	1	0	1	3	0	0	1	1	0	1	1	1	1	1	2	2	2	1	22
115	10	0	0	0	0	1	0	0	2	0	1	0	0	0	0	1	0	0	4	6	3	4	5	7	7	7	6	54
115	20	0	0	0	0	0	0	0	0	0	0	0	0	0	0	0	0	0	1	2	0	1	2	4	5	6	6	26
115	30	0	0	0	0	0	0	0	0	0	0	0	0	0	0	0	0	0	1	2	0	0	1	4	5	6	6	26
115	40	0	0	0	0	0	0	0	0	0	0	0	0	0	0	0	0	0	1	2	1	1	1	3	4	4	3	20
115	50	0	0	0	0	0	0	1	5	4	4	6	5	3	0	5	5	5	6	8	5	5	5	7	7	7	6	99

Table S4: Alkane decay rates for initial decay (0.5-year) and total 3-year decay are listed by carbon number (C15-C40). The ratio depicts the 182-d decay rate/1152-d decay rate ratio.

Carbon number	Rate constants (d^{-1})				Ratio
	182 days	Standard error	1152 days	Standard error	
15	0.0107	0.0024	0.0062	0.0011	1.73
16	0.0121	0.0027	0.0069	0.0006	1.74
17	0.0093	0.0022	0.0046	0.0005	2.04
18	0.0078	0.0018	0.0042	0.0004	1.83
19	0.0066	0.0015	0.0046	0.0004	1.44
20	0.0063	0.0013	0.0033	0.0004	1.94
21	0.0056	0.0012	0.0042	0.0004	1.31
22	0.0052	0.0012	0.0029	0.0003	1.78
23	0.0058	0.0012	0.0033	0.0004	1.79
24	0.0058	0.0012	0.0031	0.0004	1.84
25	0.0053	0.0013	0.0028	0.0004	1.87
26	0.0048	0.0011	0.0028	0.0004	1.70
27	0.0058	0.0012	0.0033	0.0004	1.78
28	0.0058	0.0012	0.0030	0.0004	1.95
29	0.0046	0.0011	0.0024	0.0004	1.94
30	0.0042	0.0015	0.0025	0.0004	1.68
31	0.0048	0.0014	0.0025	0.0003	1.95
32	0.0047	0.0011	0.0020	0.0003	2.30
33	0.0042	0.0012	0.0018	0.0003	2.36
34	0.0040	0.0014	0.0018	0.0003	2.22
35	0.0046	0.0016	0.0019	0.0003	2.41
36	0.0043	0.0016	0.0017	0.0003	2.60
37	0.0037	0.0017	0.0015	0.0003	2.45
38	0.0034	0.0016	0.0013	0.0003	2.58
39	0.0037	0.0020	0.0015	0.0004	2.44
40	0.0036	0.0051	0.0012	0.0007	2.98

Table S5. Concentrations of PAHs measured during the experiment. PAHs not listed were below the detection limit.

Day after start	Depth (cm)	biphenyl	acenaphthylene	acenaphthene	fluorene	dibenzothiopene	phenanthrene	pyrene	benzo(c)phenanthrene	chrysene	7,12-dymethylbenz(a) anthracene	benzo(b,j,k) fluoranthene	benzo(a)pyrene	benzo(g,h,i)perylene	Sum
		($\mu g\ kg^{-1}$)													
0	0	36	9	1	90	305	255	529	184	845	725	718	102	129	1476
41	10	86	6	23	33	196	237	356	143	518	484	434	687	94	7961
41	20	160	16	57	77	485	604	669	209	812	813	851	117	194	1343
41	30	35	9	30	41	289	414	556	184	709	636	716	100	151	1116
41	40	53	11	44	58	369	478	576	205	772	707	802	107	167	1226
41	50	64	12	51	59	417	542	640	203	755	714	780	106	152	1224
89	10	13	3	13	26	102	847	182	68	285	233	284	351	35	5016
89	20	8	3	12	24	87	712	174	63	254	245	247	312	32	4461
89	30	13	2	9	16	71	637	171	59	259	203	251	311	31	4367
89	40	13	2	7	13	62	516	152	52	230	175	210	271	26	3802
89	50	11	3	10	15	71	576	141	58	252	237	241	300	27	4212
131	10	14	5	12	25	110	921	232	74	331	249	301	389	37	5686
131	20	15	4	13	25	103	807	214	90	396	309	364	468	52	6429
131	30	19	3	11	19	91	748	215	78	345	270	308	410	46	5674
131	40	18	3	8	9	48	406	211	73	324	270	296	395	44	5030
131	50	12	2	7	6	59	479	157	53	230	181	318	288	29	3737
181	10	5	1	2	2	21	193	68	29	116	77	n/a	145	11	1723
181	20	6	1	1	2	8	29	82	30	122	82	n/a	153	12	1630
181	30	5	1	1	2	8	13	60	22	982	56	n/a	119	9	1278
181	40	6	1	2	2	15	63	66	26	109	66	n/a	132	11	1485
181	50	4	1	1	2	8	27	57	27	104	64	n/a	124	8	1368
235	10	37	5	14	20	137	610	394	147	465	261	337	436	27	7079
235	20	35	5	12	18	140	659	364	129	393	244	287	371	24	6225
235	30	32	4	7	12	88	349	269	109	327	185	237	309	21	4898
235	40	28	3	5	9	58	123	263	101	299	166	194	271	18	4236
235	50	27	3	5	10	60	233	242	92	275	127	168	243	15	3984
279	10	21	3	3	6	50	46	202	82	283	174	n/a	295	16	3732
279	20	30	4	2	7	45	29	342	138	449	268	n/a	460	24	5844
279	30	23	3	3	7	36	14	301	115	403	240	n/a	392	18	5186
279	40	22	2	1	4	27	14	273	128	437	258	n/a	434	20	5560
279	50	37	3	3	8	53	17	345	128	402	240	317	374	22	5521
327	10	12	2	4	6	38	171	169	69	194	86	109	166	9	2783
327	20	10	1	1	3	22	5	133	59	162	73	86	137	7	2162
327	30	11	1	1	3	27	33	165	62	172	72	94	146	8	2345
327	40	10	1	1	3	20	6	138	60	169	79	94	145	9	2256
327	50	9	1	1	3	22	8	141	60	172	84	97	150	9	2312
445	10	13	1	1	4	27	10	159	68	182	86	107	168	9	2479
445	20	13	2	1	3	21	4	144	60	165	77	85	143	8	2216
445	30	14	1	0	3	16	6	113	51	146	56	56	130	6	1912
445	40	15	1	1	3	20	5	130	61	170	73	95	151	8	2265
445	50	15	2	1	3	21	8	160	70	202	80	109	199	12	2701
735	10	27	2	1	3	22	18	176	77	211	92	127	198	12	2869
735	20	23	1	1	3	14	23	143	79	217	70	99	229	14	2873
735	30	19	2	2	6	24	24	210	95	267	134	176	262	16	3647
735	40	15	1	1	3	15	6	133	53	142	57	77	125	7	1915
735	50	14	1	1	3	13	7	156	64	169	69	86	148	8	2264
115	10	37	4	0	6	8	23	406	183	605	664	664	957	130	9137
115	20	58	3	0	5	0	1	207	124	396	247	332	776	99	5816
115	30	39	2	0	7	0	3	221	165	525	454	382	101	122	7666
115	40	38	0	0	4	1	1	273	139	511	496	499	818	86	4916
115	50	11	0	0	1	1	0	204	106	n/a	381	356	557	52	1669

Table S6: PAH decay rates for initial decay (0.5-year) and total 3-year decay are listed by carbon number (C15-C40). The ratio depicts the 182-d decay rate/1152-d decay rate ratio.

Compound	Number of rings	182 days (d^{-1})	Standard error	1152 days (d^{-1})	Standard error	Ratio
biphenyl	2	0.0122	0.0024	0.0008	0.0006	14.8
acenaphthylene	3	0.0125	0.0020	0.0024	0.0009	5.3
acenaphthene	3	0.0210	0.0026	0.0046	0.0011	4.5
fluorene	3	0.0192	0.0024	0.0042	0.0011	4.6
dibenzothiopene	3	0.0177	0.0024	0.0038	0.0007	4.7
phenanthrene	3	0.0154	0.0043	0.0073	0.0014	2.1
pyrene	4	0.0115	0.0013	0.0012	0.0008	9.3
benzo(c)phenanthrene	4	0.0107	0.0015	0.0008	0.0008	12.9
chrysene	4	0.0109	0.0023	0.0016	0.0007	6.8
7,12-dimethylbenz(a)anthracene	4	0.0127	0.0038	0.0028	0.0010	4.5
benzo(b,j,k)fluoranthene	5	0.0081	0.0121	0.0027	0.0038	3.0
benzo(a)pyrene	5	0.0112	0.0042	0.0021	0.0010	5.2
benzo(g,h,i)perylene	6	0.0143	0.0064	0.0035	0.0014	4.1
